# Quantifying the morphology of eyeballs with posterior staphyloma with Zernike polynomials

**DOI:** 10.3389/fbioe.2023.1126543

**Published:** 2023-03-09

**Authors:** Hua Rong, Lin Liu, Yuling Liu, Wanzeng Fu, He Xu, Danyang Yu, Di Wu, Bei Du, Xuejun Zhang, Bin Zhang, Ruihua Wei

**Affiliations:** ^1^ Tianjin International Joint Research and Development Centre of Ophthalmology and Vision Science, Eye Institute and School of Optometry, Tianjin Medical University Eye Hospital, Tianjin, China; ^2^ Qcraft Inc., Beijing, China; ^3^ Department of Radiology, Second Hospital of Tianjin Medical University, Tianjin, China; ^4^ College of Optometry, Nova Southeastern University, Davie, FL, United States

**Keywords:** posterior staphyloma, 3D MRI, high myopia, Zernike polynomials, eyeball morphology

## Abstract

**Purpose:** To quantify the morphology of eyeballs with posterior staphyloma (PS) with Zernike decomposition and to explore the association between Zernike coefficients with existing PS classification.

**Methods:** Fifty-three eyes with high myopia (HM, ≤-6.00D) and 30 with PS were included. PS was classified with traditional methods based on OCT findings. Eyeballs’ morphology was obtained with 3D MRI, from which the height map of the posterior surface was extracted. Zernike decomposition was performed to derive the coefficients of the 1st-27th items, which were compared between HM and PS eyes with the Mann-Whitney-U test. Receiver operating characteristics (ROC) analysis was used to test the effectiveness of using Zernike coefficients to discriminate PS from HM.

**Results:** Compared to HM eyeballs, PS eyeballs had significantly increased vertical and horizontal tilt, oblique astigmatism, defocus, vertical and horizontal coma, and higher order aberrations (HOA) (all Ps < 0.05). HOA was the most effective in PS classification with an area under the ROC curve (AUROC) value of 0.977. Among the 30 PS, 19 were the wide macular type with large defocus and negative spherical aberration; 4 were the narrow macular type with positive spherical aberration; 3 were inferior PS with greater vertical tilt, and 4were peripapillary PS with larger horizontal tilt.

**Conclusion:** PS eyes have significantly increased Zernike coefficients, and HOA is the most effective parameter to differentiate PS from HM. The geometrical meaning of the Zernike components showed great accordance with PS classification.

## 1 Introduction

Posterior staphyloma (PS) is a common complication of pathological myopia, which manifests as partial protrusion of the back of the eyeball. Retinal changes, such as retinoschisis and retinal detachment, are often observed in patients and lead to irreversible vision impairment and reduced life quality (Avila et al., 1984; Ohno-Matsui and Tokoro, 1996; Hayashi et al., 2010). As the worldwide prevalence of high myopia increases year by year, it is critical to detect PS and evaluate its severity in a timely manner (Holden et al., 2016). In the early days, the detection and classification of PS mainly depended on findings under ophthalmoscopy or imaging technology, such as optical coherence tomography or B-scan ultrasound. Curtin classified PS into ten different types based on ophthalmoscopic appearance (Curtin, 1977). Shinohara et al. characterized the PS from OCT with choroid thinning toward the staphyloma margin, inward protrusion of the sclera, and scleral reversion in the post-marginal region (Shinohara et al., 2017). These findings are often local and do not reveal much about the overall shape of the eyeball.

Three-dimensional MRI (3D MRI) can display the morphology of the eye in its entirety (Moriyama et al., 2012; Shinohara et al., 2013; Nagra et al., 2014; Wang et al., 2016). Spaide et al. described PS as a protrusion in the posterior fundus area, and its radius of curvature is smaller than that of the adjacent eye wall (Spaide, 2014). Ohno-Matsui et al. used a combination of 3D MRI and ultra-widefield fundus imaging to classify PS into six different types based on the size, shape, and location of the staphylomas (Ohno-Matsui, 2014). However, these defining signs heavily rely on human subjective judgments, including whether the curvature changes and where the choroid begins to thin. More recent studies started classifying staphyloma based on quantitative analysis of 3D MRI images. Moriyama divided staphylomas into 18 categories based on parameters derived from the 3D MRI images (Moriyama et al., 2012). Lim fitted the largest section of MRI images into an ellipse and calculated the ratio between long and short axes to quantify the asphericity (Lim et al., 2019). Ishii et al. used MRI images and Fourier transform to analyze the shape of the eyeball (Ishii et al., 2011). But these studies only utilized certain arbitrarily chosen sections. As more studies were completed, Ohno-Matsui et al., 2017 suggested that there may be more variations in the shape of staphylomas than previously thought. Therefore, it is desirable to have a method that can incorporate all the information about the eye shape.

Zernike polynomials consist of a series of polynomials orthogonal to each other (Schwiegerling and Greivenkamp, 1997; Lakshminarayanan and Fleck, 2011), which are a set of basis vectors that can describe any curved surface in the unit circle. Different terms represent specific geometric meanings, and each term’s coefficients can represent the surface shape’s component (Tango, 1977; Wang and Silva, 1980). In ophthalmology, Zernike polynomials have been used to describe corneal shape changes in keratoconus (Wacker et al., 2015; Shugyo et al., 2021). We believe that the same principles could be applied to the posterior surface of the eyeball. In this study, 3D MRI images of eyeballs with or without PS were collected, and the height maps of the posterior surface were extracted. The height maps were further decomposed into Zernike components, such as tilt, defocus, coma, astigmatism, and HOAs. We first compared the difference in Zernike coefficients between the eyeballs with and without PS. Moreover, the relationship between Zernike coefficients and existing types of PS was analyzed.

## 2 Methods

### 2.1 Patients

**FIGURE 1:**
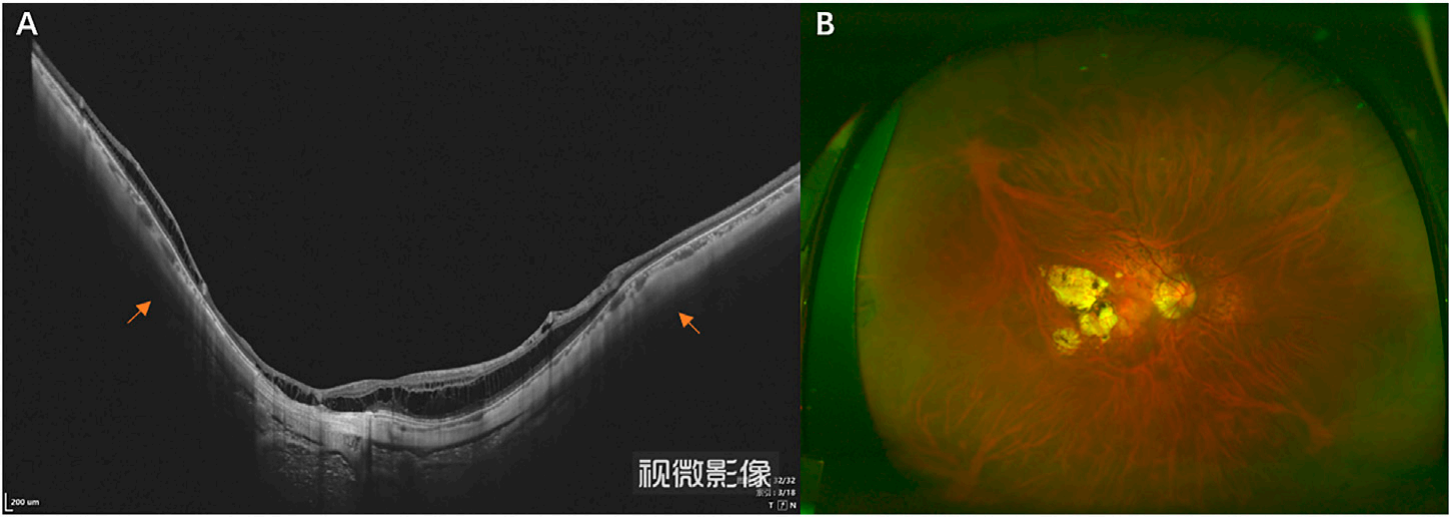
OCT **(A)** and wide-field fundus image **(B)** of an eyeball with PS. The yellow arrow indicates the choroid’s thinning and the scleral curvature change.

This prospective study included 45 participants recruited at Tianjin Medical University Eye Hospital between October 2020 and May 2022. The subjects were divided into two groups, the HM group and the PS group, respectively. HM was defined as spherical equivalent (SE)≤-6.00D, the eyeball was regular spherical or ellipsoid under 3D MRI, and there was no posterior scleral staphyloma. OCT and fundus photo examination showed no fundus lesions. A PS was defined by an outward bowing of the sclera on the OCT images, with the curvature radius of the staphyloma being smaller than the curvature radius of the surrounding sclera (Figure 1A) and with retinal atrophy evident on fundus photography (Figure 1B). There was no restriction on axial length, as staphyloma may still occur even with a short axial length (Moriyama et al., 2011; Wang et al., 2016). The diagnosis of HM or PS was made by two investigators independently, who agreed with all diagnoses made (Xu et al., 2019). The exclusion criteria were: scleral buckling, ocular trauma that could affect the eyeball shape, claustrophobia, presence of a pacemaker or intraocular metal foreign body, and systemic disease. Written informed consent was obtained from all participants. All study procedures adhered to the tenets of the Declaration of Helsinki and were approved by the ethics committee of Tianjin Medical University Eye Hospital (NO. 2020KY-04).

### 2.2 Measurements

All participants had comprehensive ocular examinations, including best-corrected visual acuity (BCVA), refractive error measurements with an auto refractometer (ARK-730; Nidek, Nagoya, Japan) without cycloplegia, axial length measurements using Lenstar LS-900 (Haag-Streit AG, Berne, Switzerland), detailed ophthalmoscopic examinations, fundus images (Optos, PLC, Dunfermline, Scotland), and OCT (VG200, SVision Imaging, Henan, China). The BCVA was measured with a chart of Landolt rings set at a distance of 5 m. For statistical analyses, the decimal BCVAs were converted to the logarithm of minimal angle of resolution (logMAR) units. The Discovery MR750 3.0T scanner (GE Healthcare, Milwaukee, WI, United States), an 8-channel phased head coil, was used for 3D MRI imaging. Axial position images were acquired using a fast-recovery-fast-spin-echo acceleration sequence (3D FRFSE-XL).

### 2.3 MRI image analysis

**FIGURE 2:**
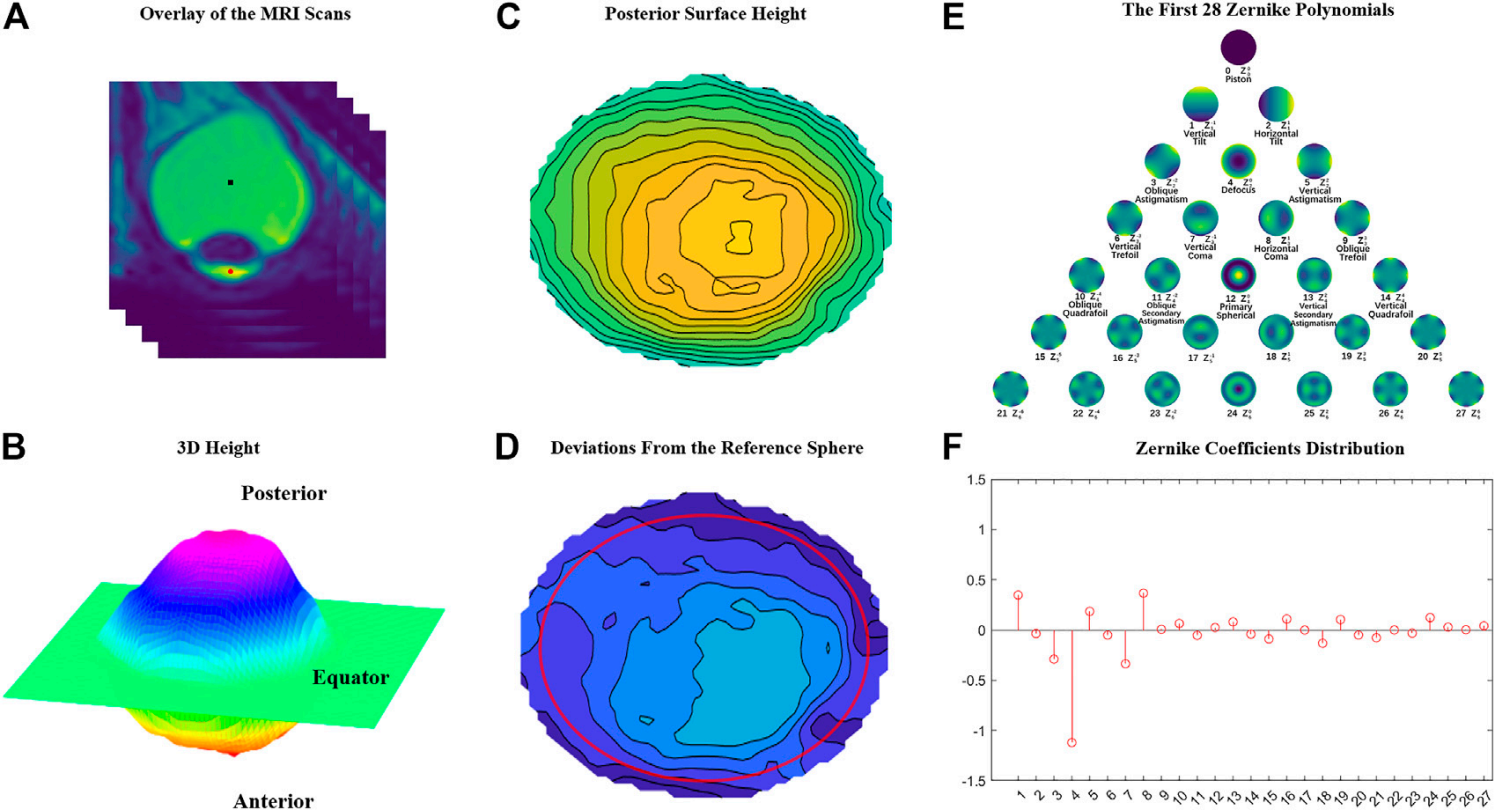
The Zernike coefficient analysis process of the posterior surface of the eyeball. **(A)** A stack of 2D MRI images is superimposed into a 3D image. **(B)** The posterior surface of a height map is extracted. **(C)** The posterior surface height map. **(D)** The residual height map after a standard sphere with a radius of 12.5 mm is subtracted from the original height map. **(E)** The first 28 Zernike polynomials derived from a residual height map. **(F)** Zernike analysis is performed on the height matrix within a radius of 11 mm (the red circle in Figure 2D).

In this study, we utilized Python3.7.9 to preprocess DICOM files of MRI images in this study. MRI images with obvious artifacts or poor quality were excluded from processing. The “breadth-first algorithm” was used to search for the boundary of the vitreous body. A search starts from an arbitrary point within the vitreous body, moving from center towards the periphery. If the contrast difference between the next point and the previous point is less than the threshold, the next point is included in the vitreous matrix; otherwise, the search is stopped. Finally, all points contained in the vitreous bodies in a slice are included in the matrix. The contrast threshold between the vitreous body and surrounding tissue was set to 1800, which allows for automatic differentiation between the two through experimentation. We determine the physical distance in every direction for each pixel based on the field of view and layer spacing of the MRI. The distance between each pixel in the same fault is 0.703 mm, and the layer spacing is 0.5 mm. In each slice, after the boundary of the vitreous chamber was extracted, the slices were stacked over in sequence, and the 3D boundary of the entire vitreous body could be extracted into a matrix (Figure 2A). The geometric center VC of the vitreous body (VC, the black point in Figure 2A) and the geometric center of the cornea (CC, the red point in Figure 2A) were also calculated. Using Rodrigues formula, we first determined the rotation matrix that can align the vector VC_to_CC with the vertical-downward direction. Then the entire eyeball is rotated with such a matrix. The libraries used in the process are pandas, numpy, math, matplotlib, mpl_toolkits, pydicom, and os from the Python ecosystem. The eyeball was divided into the front and back parts by the equator plane, which perpendicular to the axis of the VC_to_CC with the largest cross-sectional area (Figure 2B). The height of a point on the back surface was defined as its distance, parallel to the direction of the VC_to_CC vector, to the equator surface. A surface height map was derived after all points’ heights were calculated (Figure 2C). The surface height map was drawn using the mpl_toolkits.mplot3d.Axes3D.plot_surface function from a Python third-party library. To show how an eyeball’s shape deviated from a perfect sphere, a residual height map was derived by subtracting the original height map with a perfect sphere with a radius of 12.5 mm (Figure 2D) which corresponded to the median radius of the coronal plane sections of the eyeballs. Zernike analysis was performed only within the central 11 mm radius (Red circle in Figure 2D). Figure 2E is the geometric meaning of 0–28th Zernike coefficients. The coefficients of the 1st to the 27th components are shown in Figure 2F.

### 2.4 Classification of PS

We classified PS according to the classification method proposed by Ohno-Matsui (Ohno-Matsui et al., 2017), which can divide PS into six categories, namely, wide macular PS, narrow macular PS, peripapillary PS, nasal PS, inferior PS, and other types of PS.

### 2.5 Statistical analysis

The normality of data was assessed using the Kolmogorov–Smirnov test. The mean and standard deviation were calculated for SE, AL. Median and quartile were calculated for tilt, defocus, coma, astigmatism, and HOAs (6th-27th) root-mean-square (RMS) for each group. Because some Zernike terms exhibited inconsistent normality among the two groups, the two groups were compared using the non-parametric Mann-Whitney U test. Differences were considered statistically significant when the *p*-value was less than 0.05. The receiver operating characteristic (ROC) curves were built for HOAs-RMS, defocus, and coma in the classification of PS. The area under the ROC curve (AUROC) was calculated, and the cutoff value with the highest Youden indices (Youden index = sensitivity + specificity - 1) was determined. All statistical calculations were performed in IBM SPSS Statistics, version 26.0 (IBM Corp, Armonk, NY)

## 3 Results

### 3.1 Basic information

A total of 27 patients with 53 eyes in the HM group and 18 patients with 30 eyes in the PS group were included in this study. The basic information about the patients is summarized in Table 1.

**TABLE 1:** Characteristics of the study groups.

Mean ± SD	PS	HM	P
No. patients (eyes)	18 (30)	27 (53)	—
Men	8 (13)	12 (24)	—
Women	10 (17)	15 (29)	—
Age (y)	57.76 ± 12.09	48.24 ± 10.29	<0.01^*^
SE (D)	−13.90 ± 2.64	−8.50 ± 1.16	<0.01^*^
AL (mm)	29.74 ± 1.75	27.20 ± 0.76	<0.01^*^
BCVA (LogMAR)	0.66 ± 0.38	0.00 ± 0.02	<0.01^*^

*Means significant differences between the two groups using the Mann-Whitney U test.

### 3.2 Zernike components

**FIGURE 3:**
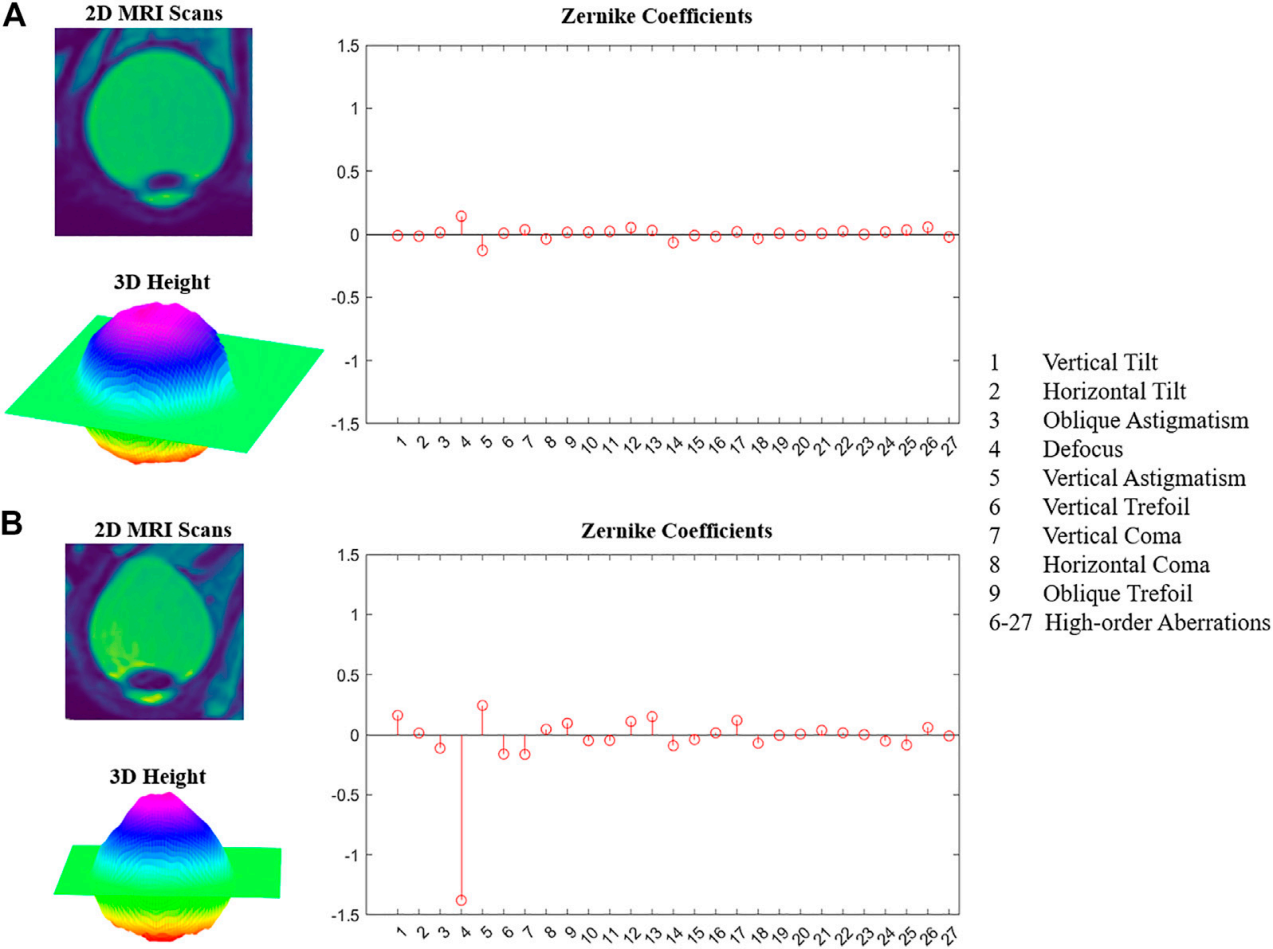
Examples of HM and PS. **(A)** HM eyeball 2D MRI image, 3D posterior surface height map, Zernike coefficients. **(B)** PS eye ball 2D MRI image, 3D posterior surface height map, Zernike coefficients.

**FIGURE 4:**
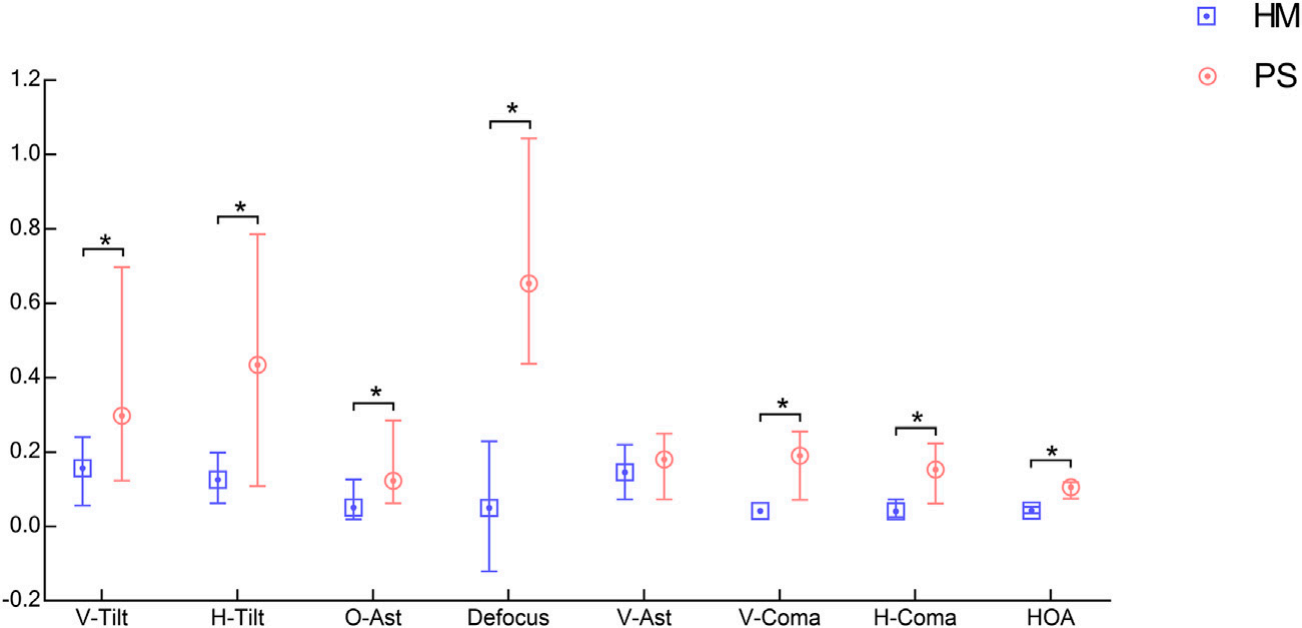
The median and quartiles values of Zernike coefficients for HM (blue) and PS (red) eyeballs.

An example of the HM eyeball (Figure 3A) and an example of the PS eyeball (Figure 3B). Compared to the HM eyeball, the posterior side of the PS eyeball was bulging out. The median and quartile values from the population data were summarized in Table 2 and illustrated in Figure 4. Mann-Whitney test results showed that vertical and horizontal tilt, oblique astigmatism, defocus, vertical and horizontal coma, and HOAs-RMS in the PS group were significantly greater than those in the HM group, and there was no significant difference in vertical astigmatism.

**TABLE 2:** Zernike coefficients and HOAs-RMS of HM and PS.

Zernike coefficients	HM	PS	Adj. P
Vertical Tilt	0.157 (0.056,0.241)	0.297 (0.124,0.698)	<0.01
Horizontal Tilt	0.126 (0.06,0.199)	0.435 (0.109,0.786)	<0.001
Oblique astigmatism	0.051 (0.019,0.127)	0.124 (0.063,0.285)	<0.001
Defocus	0.051 (-0.120,0.229)	0.654 (0.438,1.044)	<0.001
Vertical astigmatism	0.146 (0.073,0.220)	0.181 (0.073,0.251)	0.992
Vertical Coma	0.042 (0.022,0.083)	0.190 (0.072,0.256)	<0.001
Horizontal Coma	0.041 (0.026,0.073)	0.153 (0.062,0.223)	<0.001
HOAs-RMS	0.044 (0.036,0.054)	0.107 (0.076,0.120)	<0.001

### 3.3 Receiver operating characteristics analysis

**FIGURE 5:**
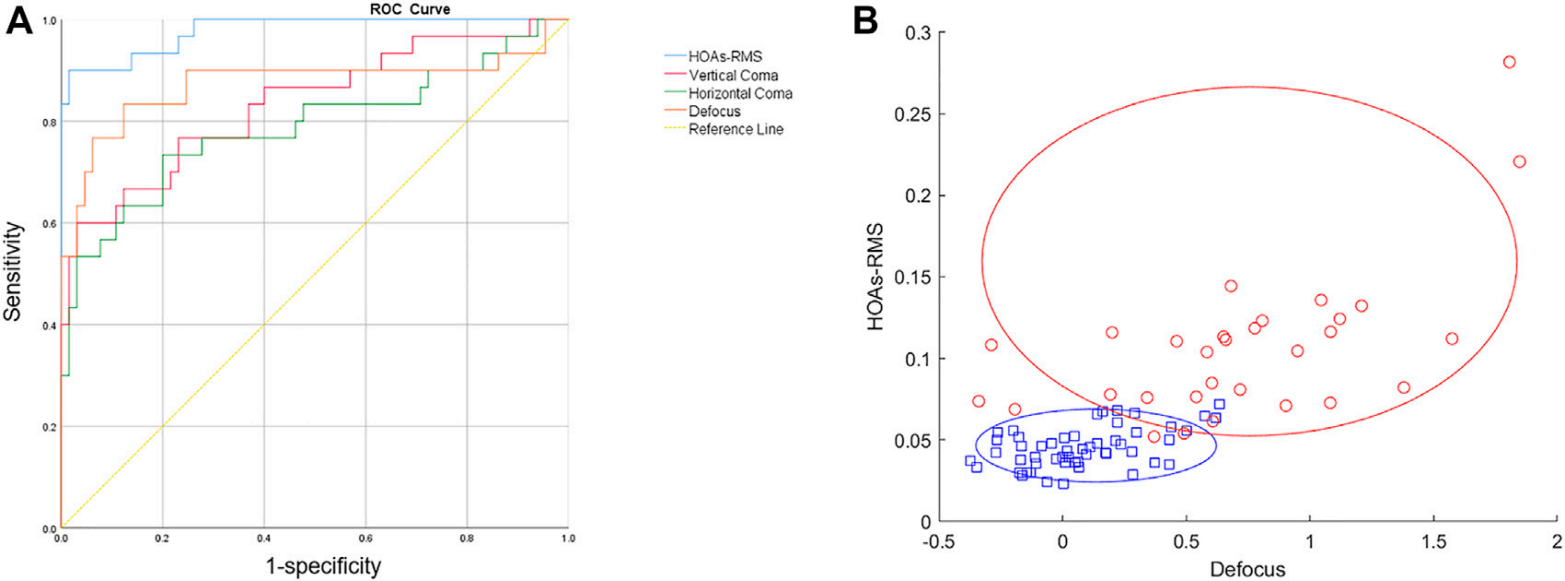
Zernike coefficients and PS classification. **(A)** ROC curves of different Zernike coefficients for PS classification. **(B)** Joint distribution of HOA and Defocus. Red: PS group. Blue: HM group.

ROC analysis was performed to see if individual Zernike coefficients could distinguish PS eyeballs from HM eyeballs. The ROC curve shows how true and false positive rate change as the criterion changes. Two completely separated populations without any overlap would push the curve to the top-left corner (0% false positive and 100% true positive) and lead to the largest AUC. Outliers from each population would increase the false positive rate and move the curve away from the top-left corner, thus creating a smaller AUC. In this study, the ROC curve was automatically generated by the SPSS software, and the AUC value was provided too. Among all parameters, HOAs-RMS has the greatest classification power with an AUROC value of 0.977. The cut-off value of 0.0686 for total ocular HOAs-RMS provided the highest Youden indices with 90% sensitivity and 98% specificity (Figure 5A). The second best parameter was the defocus, with an AUROC value of 0.864. Others are summarized in Table 3. The joint distribution of HOAs-RMS and defocus, the two parameters with the largest AUROC, illustrate the difference between HM and PS even better (Figure 5B).

**TABLE 3:** ROC analysis.

	AUROC	P	Sensitivity	Specificity	Cutoff	Youden index
HOAs-RMS	0.977	<0.001	0.90	0.98	0.0686	0.88
Defocus	0.864	<0.001	0.77	0.92	0.4500	0.69
VComa	0.827	<0.001	0.60	0.96	0.1463	0.56
HComa	0.791	<0.001	0.73	0.81	0.3312	0.54

### 3.4 PS classification and Zernike coefficients

**FIGURE 6:**
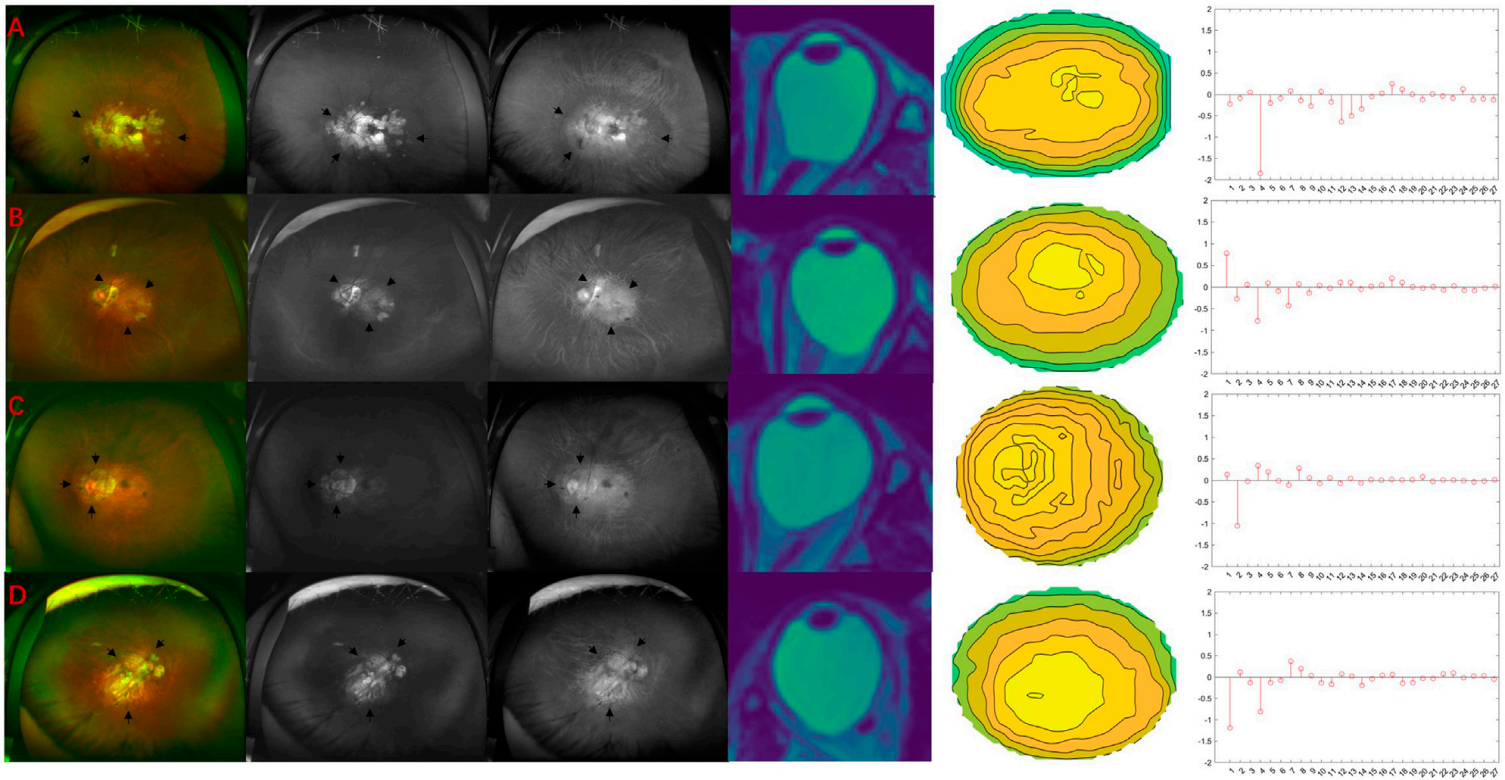
Four different types of PS. Black arrows indicate the borders of the staphyloma. **(A)** Wide macular PS. The nasal edge of macular PS exists more nasally beyond the nasal edge of the optic disc. 2D MRI tomographic image shows that the eyeball’s posterior part is blunt. The height map of the posterior part of the eyeball shows spare contour lines in the central part. Note spherical aberration (12th) is negative, indicating that the local bulge in the center is not apparent. **(B)** Narrow macular PS. The macular PS’s nasal edge is along the optic disc’s nasal edge. 2D MRI tomographic images show sharp bulges at the back of the eyeball. The height map of the back of the eyeball shows dense contour lines in the central part. Note the spherical aberration (12th) is positive, indicating that the central part is prominently raised. **(C)** Peripapillary PS. Areas of marked retinal atrophy are seen around the optic disc. The 2D MRI tomographic image shows that the eyeball protruded significantly in the direction of the optic nerve. The height map of the back of the eyeball shows denser contour lines on the left (nasal side). Note the larger horizontal Tilt (2nd term). **(D)** Inferior PS. 2D MRI tomographic image shows that the posterior pole of the eyeball is prominent, and there is no significant horizontal tilt. The contour map of the back of the eyeball shows that the prominent position is lower. Note the large vertical tilt (1st term).

In this study, we identified 19 wide macular PS (63.3%), four narrow macular PS (13.3%) eyes, three inferior PS (10%), and four peripapillary PS (13.3%). There were no nasal PS and other PS types. To illustrate the association between Zernike coefficients and the types of PS, examples of the four types of PS found in this study are shown in Figure 6, respectively.

## 4 Discussion

This study used Zernike polynomials to describe the eyeball’s posterior surface shape. The advantage of this method is that it can fully use 3D MRI information. The shape of the posterior surface of the eyeball can be reconstructed with less than 30 coefficients, and each parameter has good interpretability. This study showed that the most recognizable change in PS eyeballs was the increase of HOAs-RMS.

### 4.1 Asymmetry

Previous studies have shown that eyeballs with PS are more likely to have asymmetrical shapes (Moriyama et al., 2012; Ohno-Matsui et al., 2012). Ohno-Matsui et al. showed that the eyeballs asymmetry was significantly greater in the eyeballs with PS than in the eye without PS(Ohno-Matsui et al., 2012). Moriyama showed that PS is more likely to be located underside of the nose, which could explain the increase in oblique astigmatism in the PS group (Moriyama et al., 2012). In this study, the tilt, coma, and oblique astigmatism were significantly increased in the PS group compared with HM. These coefficients represent the degree of asymmetry in the particular meridian of the eyeball (Tango, 1977). Previous studies have shown a higher probability of fundus lesions and visual field defects in asymmetric eyeballs (Moriyama et al., 2012; Ohno-Matsui et al., 2012). Therefore, the ability to quantitatively describe the asymmetry of the eyeball has values for the clinical prediction of fundus damage. Tilt represents the inclination along the X or *Y*-axis, coma represents the asymmetry of the local height, and astigmatism means the asymmetry in the difference between the height of an axis and its orthogonal direction (Tango, 1977). Zernike analysis can distinguish the asymmetry caused by the three components, which is impossible with previous description methods.

### 4.2 Defocus and HOAs- root-mean-square

In this study, the defocus in the PS group was significantly greater than that in the HM group. Geometrically, defocus represents the overall prominence of the eyeball. The larger the defocus, the closer the eyeball was to an elongated ellipsoid (Wang and Silva, 1980). A greater defocus value indicates in PS that the overall trend of the eyeball of PS is steeper than that of the posterior surface of the eyeball of HM. In this study, ROC analysis showed that defocus was one of the key differences between PS and HM. Moriyama et al. showed that eyes with a steeper posterior surface had an increased probability of developing retinopathy (Moriyama et al., 2012; Wakazono et al., 2016), which further demonstrates the value of using the concept of defocus in evaluating eye shape.

As the HM eyeball still keep the spherical shape (Pope et al., 2017), Zernike decomposition revealed mainly low-order aberrations with few high-order aberration components. Posterior scleral PS are different in size, location, and shape (Ohno-Matsui et al., 2016). As the irregularity increases, low-order coefficients are insufficient to describe the eyeball shape fully. Therefore, in eyeballs with PS, HOAs-RMS should be more prominent. Our findings confirmed that conjecture. The values of the 6th-27th HOAs-RMS increased significantly in eyeballs with PS compared to eyeballs with HM. The combination of HOAs-RMS and defocus can distinguish PS from HM even better.

### 4.3 PS classifications

Different types of PS can cause retinal lesions in different parts, resulting in varying degrees of vision or visual field loss (Ohno-Matsui, 2014; Verkicharla et al., 2015; An et al., 2021; Lee et al., 2021). Many researchers have tried to propose a classification method for PS. Curtin divided PS into ten categories in 1977 (Curtin, 1977). On this basis, Ohno-Matsui divided PS into six categories, which is the most commonly used PS classification method (Ohno-Matsui et al., 2017). This study observed the proportion of different types of PS and the distribution difference of Zernike coefficients. The proportion of PS classification is basically consistent with previous research (Nagaoka et al., 2011; Ohno-Matsui et al., 2017). Wide macular PS accounted for the largest proportion, reaching 63.3%. The Zernike coefficients distribution of wide macular PS is characterized by large defocus. This is due to the wide macular PS eyeball showing a broad protrusion at the back, such as in Figure 6A. Although the posterior part of the eyeball is convex overall, the posterior pole is blunt. Compared with a perfectly spherical surface, the central part is relatively concaved. Therefore, the spherical term, which indicates the central local bulge is negative. In contrast, the narrow macular PS showed a greatly increased spherical term, such as in Figure 6B. This is because of the narrow central protrusion at the posterior pole. The eyeball of the peripapillary PS mainly showed a significant increase in horizontal tilt, which represented a tilt of the nose, while the optic nerve was located on the nasal side. The vertical tilt of the inferior PS was significantly reduced, which represented an increase in the inferior tilt. These results show the consistency between the Zernike coefficients and the actual eyeball geometry. In previous studies, eyeballs with an irregular or difficult-to-describe shape were defined as other classes, including double-edged staphyloma, etc (Ohno-Matsui et al., 2017). This also reflects that the traditional method has difficulty describing eyeballs in detail. Zernike analysis has the advantage of describing complex eyeball shapes in more detail and helps in the future automatic objective classification of PS.

## 5 Limitations

Firstly, the number of PS patients included in this study is relatively small. Therefore, we did not find the nasal PS and other PS. Secondly, although the eyeball shape was quantitatively described in this study, the relationship with the degree of fundus damage in patients was not examined. In future studies, these should be achieved.

## 6 Conclusion

The Zernike polynomial can describe the morphology of the posterior eyeball well. The defocus, tilt, oblique astigmatism, coma, and HOAs-RMS of PS all had significant changes compared with HM. HOAs-RMS had the best effect in distinguishing PS from HM. In various types of PS, the changes in Zernike coefficients matched well with the morphological changes.

## Data Availability

The raw data supporting the conclusion of this article will be made available by the authors, without undue reservation.
